# Antibacterial Porous Coaxial Drug-Carrying Nanofibers for Sustained Drug-Releasing Applications

**DOI:** 10.3390/nano11051316

**Published:** 2021-05-17

**Authors:** Xin Chen, Honghai Li, Weipeng Lu, Yanchuan Guo

**Affiliations:** 1 Key Laboratory of Photochemical Conversion and Optoelectronic Material, Technical Institute of Physics and Chemistry, Chinese Academy of Sciences, Beijing 100190, China; chenxin@mail.ipc.ac.cn (X.C.); lhh@mail.ipc.ac.cn (H.L.); 2 University of Chinese Academy of Sciences, Beijing 100049, China

**Keywords:** coaxial electrospinning, porous nanofibers, PCL/PLA, antibacterial activity, drug release, phase separation

## Abstract

The phenomenon of drug burst release is the main problem in the field of drug delivery systems, as it means that a good therapeutic effect cannot be acheived. Nanofibers developed by electrospinning technology have large specific surface areas, high porosity, and easily controlled morphology. They are being considered as potential carriers for sustained drug release. In this paper, we obtained polycaprolactone (PCL)/polylactic acid (PLA) core-shell porous drug-carrying nanofibers by using coaxial electrospinning technology and the nonsolvent-induced phase separation method. Roxithromycin (ROX), a kind of antibacterial agent, was encapsulated in the core layer. The morphology, composition, and thermal properties of the resultant nanofibers were characterized by scanning electron microscopy (SEM), attenuated total reflection Fourier transform infrared (ATR-FTIR) spectroscopy, differential scanning calorimetry (DSC) and thermogravimetry analysis (TGA). Besides this, the in vitro drug release profile was investigated; it showed that the release rate of the prepared coaxial porous nanofibers with two different pore sizes was 30.10 ± 3.51% and 35.04 ± 1.98% in the first 30 min, and became 92.66 ± 3.13% and 88.94 ± 1.58% after 14 days. Compared with the coaxial nonporous nanofibers and nanofibers prepared by uniaxial electrospinning with or without pores, the prepared coaxial porous nanofibers revealed that the burst release was mitigated and the dissolution rate of the hydrophobic drugs was increased. The further antimicrobial activity demonstrated that the inhibition zone diameter of the coaxial nanofibers with two different pore sizes was 1.70 ± 0.10 cm and 1.73 ± 0.23 cm, exhibiting a good antibacterial effect against *Staphylococcus aureus*. Therefore, the prepared nanofibers with the coaxial porous structures could serve as promising drug delivery systems.

## 1. Introduction

At present, the problem of burst release during drug action should not be ignored. Drug burst release requires a high dose and multiple administrations, produces toxic and side effects, and fails to have a good therapeutic effect [1]. Therefore, the sustained release of drugs is crucial in the drug delivery system.

Due to its low cost and easy operation, electrospinning technology stands out among many nanofiber preparation technologies and can prepare different forms of nanofibers [2,3,4]. The prepared nanofibers have large specific surface areas, high porosity, and similar structures to the cytoplasmic matrix, which improves the bioavailability of substances. The electrospun nanofibers are known as favorable drug carriers [5,6,7]. Therefore, as a simple and universal method for manufacturing drug delivery systems, electrospinning technology has attracted more and more attention in the field of sustained drug release [8].

The functional parameters of drug release systems can be controlled by adjusting the morphology and specific surface areas of nanofibers [3]. The encapsulation conditions of drugs are related to their nanofibrous structures. When uniaxial electrospinning is used to prepare drug-carrying nanofibers, drugs are easily exist on the surface of the nanofibers, leading to instant drug release [9]. Electrospinning technology can also prepare different kinds of nanofiber structures, such as a core-shell structure and a porous structure, flexibly. Compared with conventional uniaxial electrospinning nanofibers with a smooth surface and solid heart, these nanofibers have larger specific surface areas and excellent physicochemical properties, which are favorable for application in tissue engineering, drug delivery, and other fields [10,11].

Coaxial structure nanofibers have been used in the research of drug release materials. The coaxial electrospinning nanofibers can encapsulate the drugs in the core layers, and the shell layers act as barriers for the release of the drugs. He and Huang et al. prepared core-shell tetracycline hydrochloride (TCH)/polylactic acid (PLLA) nanofibers. The experiment’s results showed that the release rate of TCH (3 wt%)/PLLA (10 wt%) uniaxial blended nanofibers was 30% in the first 8 h, while the release rate of coaxial TCH (10 wt%)/PLLA (10 wt%) fibers was only 22.9% after 144 h. The core-shell nanofibers inhibited the initial release and promoted the continuous release of the drugs [12]. Compared with uniaxial electrospinning, coaxial nanofibers store and control the release of drugs, protect the biological activity of the drugs from the environment, achieve more lasting drug release, and have a better therapeutic effect [9,13,14,15,16,17]. In the process of coaxial electrospinning, changing the shell layer flow rate, the shell solution concentration, and the core layer flow rate can produce shell layers of different thicknesses and thus control the release rate [14,18]. Coaxial nanofibers can also be combined with nanoparticle materials to slow down the burst release behavior of drugs [19]. Generally speaking, nanofibers with core-shell structures are promising platforms for drug delivery.

The porosity of the nanofibrous scaffold and the porosity of a single nanofiber affect the drug release effects [20]. High surface areas and porosity are the main advantages of nanofibers as drug delivery systems, which are conducive to cell anchoring and modify the wetting process, adsorption/absorption, and release behavior of drugs [2,14]. Porous nanofibers can be formed by changing the environment and solvent selection, and by using sacrificial components [21,22,23]. Han and Jiang et al. used 50% high humidity to prepare porous PLLA nanofibers with different asiatic acid (AA) contents to increase specific surface areas. The prepared porous nanofibers promoted cell adhesion, shortened the drug release cycle, and accelerated the early healing of diabetic wounds [24]. Lanno and Ramos et al. prepared porous microfiber scaffolds under high humidity conditions which were more beneficial to the attachment and growth of fibroblasts than nonporous fibers [25]. Sharifi and Sooriyarachchi et al. obtained porous and nonporous nanofibers from PCL solutions with different solvents, respectively. The experiment results proved that porous microfibers were better than nonporous microfibers in the drug release behavior [3]. The drug release can be regulated by changing the porosity of the nanofibers, and drug dissolution can be increased by the porous structure.

The ideal drug carriers should be compatible, and drug release behavior based on biodegradable materials is favorable in controlling sustained drug release [1,19]. PCL and PLA are both biocompatible, biodegradable, and non-toxic synthetic polymer materials. PCL has good biomechanical properties, but its degradation rate is low. Meanwhile, PLA has a high degradation rate and melting point, but weak mechanical properties. Coaxial electrospinning enhances the physicochemical properties of the nanofibers without considering the miscibility. As a shell material, PLA can support the proliferation and attachment of various cells [15]. ROX is a kind of macrolide antibiotic, with a good antibacterial effect on Gram-positive bacteria such as *Staphylococcus aureus.* The matching of hydrophobic drugs with the hydrophobic materials used is more beneficial to the sustained release of drugs [26].

In this study, we used the electrospinning technique and nonsolvent-induced phase separation method to prepare coaxial porous PCL/PLA drug-loaded nanofibers. ROX was encapsulated in the core layer. The physicochemical properties of the nanofibers were characterized by different methods. Compared with the coaxial nonporous nanofibers and nanofibers prepared by uniaxial electrospinning with or without pores, the obtained coaxial porous nanofibers not only slowed down the drug burst release but also increased the dissolution of hydrophobic drugs. Besides this, the prepared nanofibers had good in vitro antibacterial activity against *Staphylococcus aureus*. The results indicate that the coaxial porous nanofibers obtained in this paper are promising drug release materials.

## 2. Materials and Methods

### 2.1. Materials

The polycaprolactone (PCL, with an average molecular weight of 80,000) was purchased from Perstorp (Shanghai, China) Chemical Products Trading Co., Ltd. (Shanghai, China). The polylactic acid (PLA, with an average molecular weight of 100,000) was obtained from Hisun Biological Materials Co., Ltd. (Taizhou, China). The 2,2,2-trifluoroethanol (TFE) was purchased from Macklin Biochemical Co., Ltd. (Shanghai, China). *Staphylococcus aureus* (*S. aureus*) was obtained from the China General Microbiological Culture Collection Center (Beijing, China). The chloroform (CF) was purchased from Zhiyuan Chemical Reagent Co. Ltd. (Tianjin, China). The dimethyl sulfoxide (DMSO) was purchased from Chemical Works (Beijing, China). The phosphate buffer solution (PBS, powder, 0.01 M, pH = 7.2–7.4) was obtained from Solarbio Science & Technology Co., Ltd. (Beijing, China). The technical agar powder (TAP) was purchased from Huankai Microbial Sci. & Tech, Co., Ltd. (Guangzhou, China). The tryptone soy broth (TSB) was supplied by Aoboxing Biotechnology Co., Ltd. (Beijing, China). All of the chemical reagents were of analytical grade and were utilized as received.

### 2.2. Preparation of PCL/PLA Nanofibers

**Figure 1:**
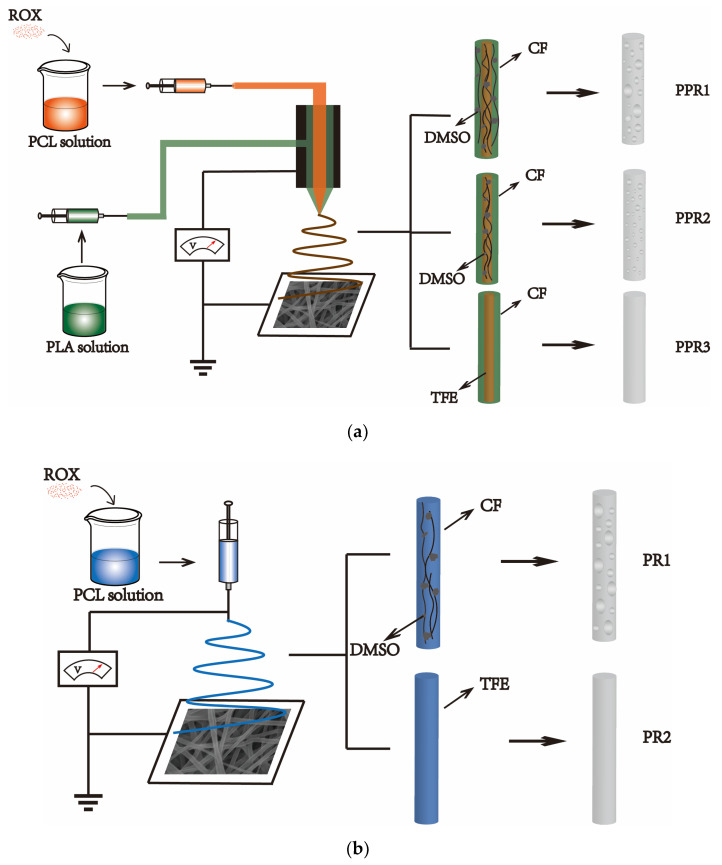
Schematic illustrations of the fabrication of the nanofibers by (**a**) coaxial electrospinning and (**b**) uniaxial electrospinning. PPR1, PPR2, and PPR3 represent the Roxithromycin (ROX)-loaded PCL/PLA nanofibers with various solvent types, and PR1, PR2 represent the ROX-loaded PCL nanofibers created by uniaxial electrospinning with different solvents. Polycaprolactone (PCL) solution and polylactic acid (PLA) solution were used as the core layer and the shell layer of the coaxial electrospinning, respectively. The solvents volatile speeds were chloroform (CF) > Trifluoroethanol (TFE) > dimethylsulphoxide (DMSO). The presence of nonsolvent DMSO, which is difficult to volatilize, and the volatile solvent CF can induce nonsolvent-induced phase separation and generate the porous structure.

For the preparation of PCL/PLA coaxial nanofibers, two kinds of PCL polymer solutions with a concentration of 10% (*w*/*v*) were prepared by adding PCL into TFE and 90/10 (*v*/*v*) CF/DMSO under constant stirring until it dissolved completely, respectively. Then, the weighed ROX powder was added to the PCL solution. The solution was stirred until the ROX was dissolved completely. The concentration of the ROX was 15 mg/mL with respect to the PCL solution. Two kinds of shell solution with a concentration of 8% (*w*/*v*) were prepared by dissolving PLA into CF and 90/10 (*v*/*v*) CF/DMSO under constant stirring until it completely dissolved, respectively. All of the solutions were prepared at room temperature. As shown in Table 1, the polymer solution was added into 20 mL syringes with different combinations. The electrospinning was performed with a coaxial needle, of which the shell diameter was 1.45 mm and the core diameter was 0.6 mm. The distance between the drum collector and the needle tip was 13 cm, the applied voltage was 16 kV, and the drum collector rotation speed was 100 rpm. The PCL/PLA nanofibers were spun with a flow rate of the shell solution of 0.2 mL/h and a feeding rate of the core solution of 0.1 mL/h. For the preparation of the PCL nanofibers by uniaxial electrospinning, the flow rate of the shell solution in the coaxial electrospinning was reduced to 0 mL/h. The electrospinning experiments were carried out at room temperature, and the relative humidity was 20–23%. As illustrated in Table 1, PPR1, PPR2, and PPR3 represent the ROX-loaded PCL/PLA nanofibers with various solvent types, and PR1, PR2 represent the ROX-loaded PCL nanofibers by uniaxial electrospinning with different solvents. Additionally, P1 and P2 represent PR1 and PR2 without drugs, respectively. The schematic illustrations are shown in Figure 1.

### 2.3. Characterization

The microstructure of the nanofibers was characterized by an S-4800 scanning electron microscope (SEM, Hitachi, Tokyo, Japan). The functional groups of the nanofibers were recorded by Fourier Transform Infrared (FTIR, Excalibur 3100, Varian, Palo Alto, CA, USA) Spectroscopy in ATR mode, and the wavelength range was 4000–600 cm^−1^. The functional groups in the ROX powers were also characterized by FTIR in the scanning range of 4000–400 cm^−1^. Differential Scanning Calorimetry (DSC, NETZSCH, Selb, Freistaat Bayern, Germany) was used to investigate the thermal properties of the samples. The samples were equilibrated at 0 °C and then heated to 200 °C at a heating rate of 10 °C/min in a nitrogen atmosphere. The thermal stability of the samples was also analyzed by thermogravimetry (TGA, NETZSCH, Selb, Freistaat Bayern, Germany) in a nitrogen atmosphere, and the sample was heated from 0 °C to 600 °C at a heating rate of 10 °C/min.

### 2.4. Antibacterial Evaluation

The in vitro antimicrobial activities of the nanofibers against *S. aureus* were determined by the inhibition zone method. TSB and TAP were used as the culturing media. First, the nanofibers were cut into circles 0.5 cm in diameter. Then, 500 μL *S. aureus* suspension was spread onto an agar plate. Finally, samples of the rounded nanofibers were placed under an ultraviolet lamp for 30 min to be sterilized, pasted onto the agar plates, and incubated at 37 °C. The bacterial growth on the plate was visualized directly, and the diameter of the inhibition zone was measured. All of the experiments were performed in triplicate.

### 2.5. Drug Release Studies

The calibration curve of ROX was prepared by using solutions in six different concentrations: 0.015 g/L, 0.03 g/L, 0.05 g/L, 0.075 g/L, 0.1 g/L and 0.15 g/L. For the drug release test, nanofibers with a certain weight were immersed into the appropriate amount of PBS and then incubated at 37 °C in a shaking incubator (CHA-S, Shenglan, Changzhou, China) with mild shaking. At predetermined time intervals, 3 mL of the soaking solution was withdrawn for ROX detection to determine the amount of drug released, and was replaced by another 3 mL of fresh PBS to maintain a constant volume. The released ROX drugs were assessed at a wavelength of 202 nm using a UV-visible spectrophotometer (UV-vis, Puxi, Beijing, China). The concentration of the released ROX was back-calculated from the predetermined calibration curve of the ROX. The percentage of the drug released was calculated based on the initial weight of the drug incorporated into the nanofibers. The experiments were repeated three times for each nanofiber. The data are expressed as mean ± standard deviation.

The accumulative percentage of the drug released from the nanofibers was calculated using the following equation:

(1) $$C\left(\%\right)=\frac{C_{n}\times V_{0}+\sum_{i=1}^{n-1}C_{i}\times V}{Q_{0}}\times 100$$

where *V_0_* is the volume of the medium in the tubes (mL), *V* is the volume of the withdrawn solution for ROX detection (mL), *C_n_* is the drug concentration determined at No. *n* (g/L), *C_i_* is the drug concentration determined at No. *i* (g/L), and *Q_0_* is the total amount of ROX in the nanofibers (mg).

## 3. Results and Discussion

### 3.1. Characterization of the Drug-Loaded PCL/PLA Nanofibers

#### 3.1.1. Morphological Properties

**Figure 2:**
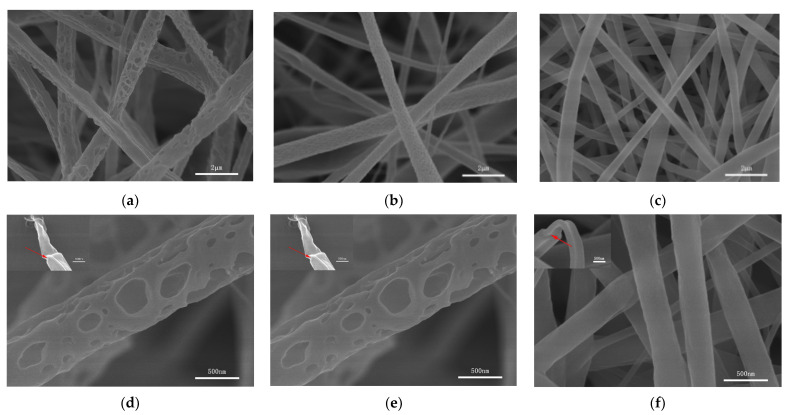
SEM images of the coaxial nanofibers: (**a**,**d**) PPR1, (**b**,**e**) PPR2, (**c**,**f**) PPR3. The red arrows highlight the coaxial structure.

In this paper, coaxial porous nanofibers were prepared by nonsolvent-induced phase separation. The presence of nonsolvents caused the thermodynamic instability of the polymer solution and initiated phase separation. Compared with other phase separation mechanisms, nonsolvent-induced phase separation can be controlled, and can provide repeatable results [27]. As seen in Figure 2, the surfaces of the nanofibers with different morphologies were uniform and beadles, and there were no drug particles found on the surfaces of the nanofibers.

In the coaxial porous structure shown in Figure 2a,d, different sizes of pores were distributed on the surfaces of nanofiber PPR1. CF/DMSO was used as the solvent for both the core layers and the shell layers. The same solvent systems can reduce the influence of interfacial tension on the electrospinning process. CF is a volatile solvent, while DMSO is less volatile and a nonsolvent of PCL and PLA. In the process of jet flow, the CF in both layers is volatilized first, which reduces the temperature of the polymer solution, promoting the instability of the jet flow, and generating phase separation, forming polymer aggregation areas and nonsolvent aggregation areas. Then, the nonsolvent DMSO is volatilized in both layers, forming holes on the surfaces of the nanofibers. In Figure 2b,e, the surfaces of the PPR2 were covered with smaller pores. During the formation process, the solution of the core layer—which used CF/DMSO as the solvent—changed, as mentioned above. Meanwhile, only one solvent, CF, was used in the shell solution. CF volatilized rapidly with the nanofibers which were cured quickly. In this case, the shell had a degree blocking effect on the volatilization of the DMSO in the core layer, and the porous structure could not be formed as shown in Figure 2a,d [11]. At the same time, we should know that the volatilization of CF made the surrounding temperature drop, and the water vapor in the surrounding air condensed on the surfaces of the nanofibers. With the gradual drying and solidification of the nanofibers, the condensed water volatilization formed uniform small pores. The common and easy to operate methods for preparing porous nanofibers by electrospinning include phase separation and the breath figure technique [28]. It can be speculated that the formation of coaxial porous nanofibers was the result of the combined action of nonsolvent-induced phase separation and the breath figure technique. Compared with phase separation, this process of the breath figure technique occurred relatively slowly [15]. In the nonporous coaxial structure shown in Figure 2c,f, nonsolvent DMSO was no longer used in the shell or core layers, and so PPR3 with smooth surfaces was obtained. Although both TFE and CF were volatile solvents, the high boiling point of TFE and the low humidity conditions of this experiment were not conducive to the formation of nanofiber pores, resulting in the formation of PPR3 with a smooth surface and compact interior.

**Figure 3:**
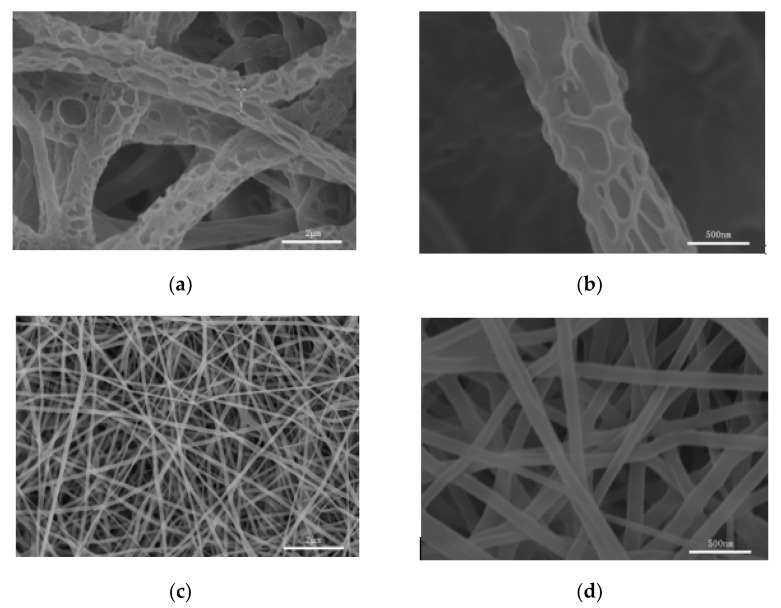
SEM images of nanofibers (**a**,**b**) PR1, (**c**,**d**) PR2, as prepared by uniaxial electrospinning.

In addition to the above, as shown in Figure 3, this paper used uniaxial electrospinning to prepare porous nanofiber PR1 and nonporous nanofiber PR2. The coaxial electrospinning could be changed to uniaxial electrospinning by adjusting the flow rate of the shell layer to 0 mL/h. CF/DMSO was used as a solvent for PR1 and TFE was used as a solvent for PR2. The diameter of the nonporous nanofibers was smaller than that of the corresponding porous nanofibers due to the fact that high-conductivity TFE was used as the solvent [22].

#### 3.1.2. FTIR Analysis

**Figure 4:**
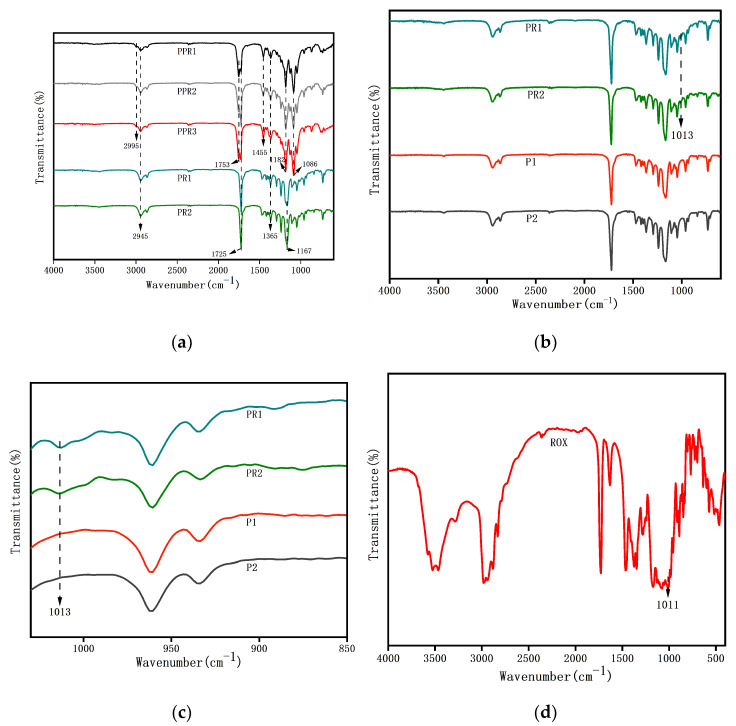
FTIR spectra of (**a**–**c**) drug-loaded nanofibers PPR1, PPR2, PPR3, as prepared by coaxial electrospinning; drug-loaded nanofibers PR1, PR2 and drug-free nanofibers P1, P2, as prepared by uniaxial electrospinning; and (**d**) ROX. Figure 4c is a partial enlargement of Figure 4d.

FTIR was tested to ascertain the chemical composition of the nanofibers. As shown in Figure 4a, the spectra of all of the kinds of nanofibers present three characteristic peaks at 1365, 1725, and 2945 cm^−1^, corresponding to the bending vibration of C-H, the stretching vibration of carboxyl (C = O), and the stretching vibration of C-H of PCL, respectively. Five new peaks exhibited in PPR1, PPR2, and PPR3 containing PLA were assigned to the stretching vibration peak of C-H at 2995 cm^−1^, the stretching vibration peak of carboxyl (C = O) at 1753 cm^−1^, the bending vibration peak of C-H at 1455 cm^−1^, and the asymmetric stretching vibration of C-O-C at 1086 cm^−1^ and 1182 cm^−1^, respectively. Besides this, we can see that the asymmetric stretching vibration of C-O-C at 1167 cm^−1^ that appeared at PR1 and PR2 was missing, and the change of the characteristic peaks was caused by the blocking effect of the C-O-C of PLA. Because of the small amount of ROX in the solution, there were no evident characteristic peaks of ROX in the spectra. What we need to know is that the uniaxial electrospinning used the same solution as the core layer of the coaxial electrospinning, so we compared nanofibers prepared by uniaxial electrospinning with and without drugs in order to determine whether ROX was loaded into the nanofibers (Figure 4b). As presented in Figure 4c, we enlarged the local spectrum of Figure 4b. The characteristic peaks at 1013 cm^−1^ of PR1 and PR2 were attributed to the C–N stretching vibration of ROX (Figure 4d) compared with the spectrum of the drug-free P1 and P2, indicating that the ROX was encapsulated into the PCL nanofibers successfully.

#### 3.1.3. Thermal Characteristics

**Figure 5:**
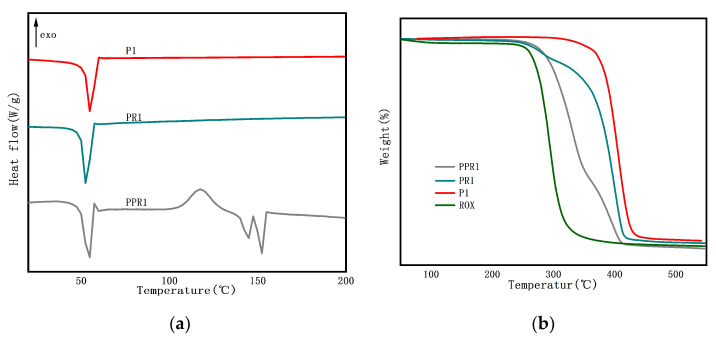
(**a**) DSC curve and (**b**) TGA curve of nanofibers PPR1, PR1, P1 and ROX.

We investigated the thermal characteristics of porous coaxial nanofibers by DSC and TGA. PCL is a semi-crystalline polyester. In Figure 5a, the endothermic peak (T_m_) of P1 was 54.81 °C, which corresponds to the melting temperature of PCL. After the addition of ROX, the melting peak shifted slightly, to 52.41 °C, indicating weak interactions between ROX and PCL. The melting point of ROX is around 120 °C. It should be noted that PR1 didn’t reflect the melting peak of the drugs, and ROX has good solubility in the solvents used, indicating that the drugs existed in the nanofibers in their non-crystalline state [18]. It was seen from the PPR1 that the crystallization temperature (T_c_) of PLA was 117.42 °C. PPR1 also showed double melting peaks at 144.88 and 152.36 °C that were related to the two different crystalline morphologies of PLA. There might be hydrogen bonding among ROX, PLA, and PCL, which reduced the thermal stability of the drug-loaded nanofibers [29]. In Figure 5b, as shown in P1, PCL had good stability, with an extrapolated onset decomposing temperature (T_e_) of 380.14 °C. ROX was sensitive to heat, and the T_e_ occurred at 272.53 °C. The degradation of PR1 began at 252.12 °C and reached the maximum decomposition rate at 401.06 °C as the temperature increased. The PPR1 has the maximum decomposition rate, at 332.46 °C, which is mainly attributed to PLA degradation. The results showed that the addition of ROX reduced the thermal stability of the nanofibers.

### 3.2. Antibacterial Activity

**Figure 6:**
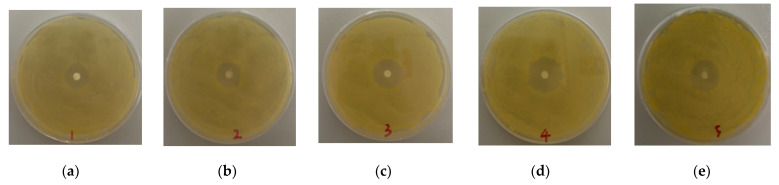
Photographs of the antibacterial test of (**a**) PPR1, (**b**) PPR2, (**c**)PPR3, (**d**) PR1 and (**e**) PR2 after incubation for 24 h at 37 °C.

Bioactive drugs can be released and remain bioactive after release from nanofibers [14]. The ROX encapsulated in the nanofiber attached to the culture medium spread circularly around the round nanofibers, forming an inhibition zone. As shown in Figure 6a–e, the nanofibers were cut into 5 mm circles of equal size. Due to the higher drug content, the nanofibers PR1 and PR2 prepared by uniaxial electrospinning had larger inhibition zones, with diameters of 2.20 ± 0.10 and 2.17 ± 0.12 cm, respectively. Meanwhile, PPR1, PPR2, and PPR3 were 1.70 ± 0.10, 1.73 ± 0.23, and 1.87 ± 0.15 cm in diameter, respectively, which were smaller than PR1 and PR2. All of the nanofibers with different structures had a good antibacterial effect against *Staphylococcus aureus*, and ROX maintained good antibacterial activity. As the drugs diffused to a larger volume, the efficacy of the drugs decreased and the diameter of the bacteriostatic zones reduced [30]. In this experiment, the diameters of the inhibition rings for all of the nanofibers remained unchanged on a scale of 14 days. However, nanofibers with a weak burst release effect and sustained release would have a better antibacterial effect.

### 3.3. In Vitro Drug Release

#### 3.3.1. Standard Curve

**Figure 7:**
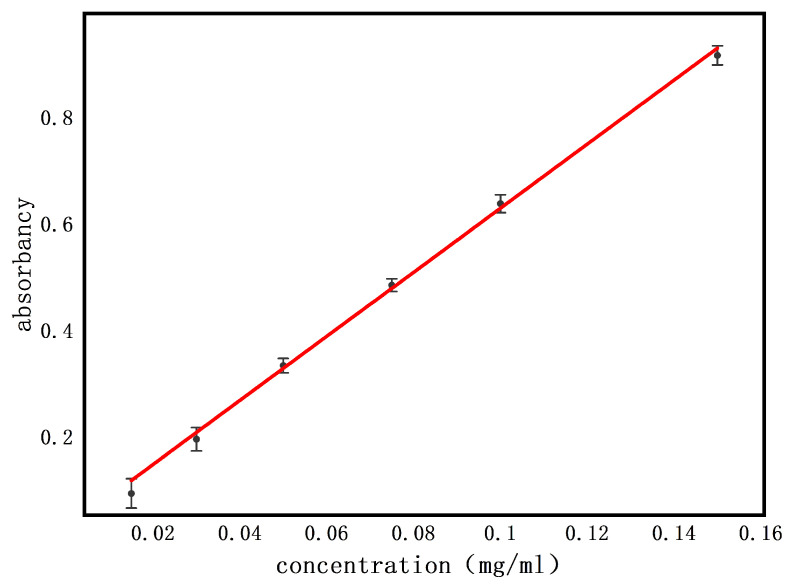
The standard curve of ROX.

The standard curve of ROX is shown in Figure 7. The maximum absorbance of ROX measured by the UV-vis spectrophotometer was 202 nm. The standard curve obtained was y=6.01775×x+0.026, and it showed linearity and acceptability with R^2^ ≥ 0.99.

#### 3.3.2. Drug Release Profile

**Figure 8:**
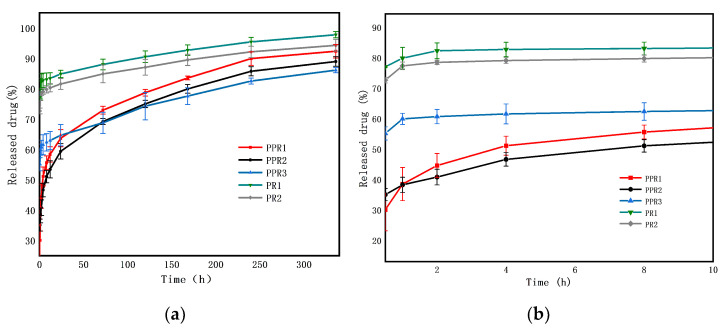
ROX release profiles of coaxial nanofibers PPR1, PPR2 and PPR3, and nanofibers PR1 and PR2, as prepared by uniaxial electrospinning (**a**) for 14 days, and (**b**) for 10 h.

The drug release behavior of drug-loaded nanofibers is affected by the morphology of the nanofibers [15]. A promising drug release system should not only meet the drug release capacity but also be able to control the drug release rate [13]. The drug release profile of the nanofibers in vitro was tested for 14 days (Figure 8). When the nanofibers were in a simulated humoral environment, the drugs immediately began to release [4]. For the nanofibers prepared by uniaxial electrospinning, the drugs dissolved quickly on or near the surface of the nanofibers, resulting in an evident burst release. In contrast, the coaxial nanofibers controlled the instant release of the drug, showing continuous release. Drug release mechanisms from coaxial nanofibers should consider three factors: diffusion, degradation, and shell thickness [14]. Due to the slow degradation of PCL and the controlled flow rate of the shell being consistent, the difference of the release effect caused by diffusion was mainly considered in this experiment. The initial release was the rapid diffusion of the drugs deposited on the nanofiber surface, while the slow release was mainly the diffusion of the drug through the polymer matrix.

As shown in Figure 8a, the total amount of the released ROX from the prepared PPR1, PPR2, PPR3, PR1, and PR2 was 30.10 ± 3.51%, 35.04 ± 1.98%, 55.14 ± 2.17%, 77.00 ± 0.27%, and 72.66 ± 0.92% in the first 30 min, respectively, and reached 92.66 ± 3.13%, 88.94 ± 1.58%, 86.08 ± 0.85%, 97.78 ± 1.05% and 94.29 ± 1.94% after 14 days, respectively. The accumulative delivery amount of the nanofibers prepared by uniaxial electrospinning was higher than that of the coaxial nanofibers, and the accumulative delivery amount of porous nanofibers was higher than that of nonporous nanofibers after 14 days, which is PR1 > PR2 > PPR1 > PPR2 > PPR3. The initial stage of drug release is shown in Figure 8b. There is an obvious burst release for nanofibers PR1 and PR2. Because of the smaller diameter and higher specific surface areas, the release rate of PPR3 was faster than the other coaxial nanofibers PPR1 and PPR2. The pore size of PPR1 was larger than that of PPR2, but the larger pore size increased the hydrophobicity of PPR1 and inhibited the release of the drugs at the initial stage of release, meaning a faster release for PPR2. With the progress of the drug release, due to the high specific surface areas and porosity of PPR1 and PPR2, the PBS solution easily penetrated the core phase, and the release degree surpassed that of PPR3 gradually. In general, the nanofibers had a high drug loading rate. The burst release of drugs at the beginning was conducive to the rapid relief of patients’ symptoms, and the continuous release of the remaining drugs optimized the treatment [18]. The drug release in the polymer combined solid diffusion and limited desorption; only the drugs on the nanofiber’s surface and pores’ surfaces released, but the drugs encapsulated inside the nanofiber were not released fully within the current experimental time scale, so the complete release was not achieved [31].

#### 3.3.3. Drug Release Mechanism

At present, the commonly used drug release kinetics models include the zero-order model, the first-order model, the Higuchi model, and the Ritger–Peppas model, which are used to explore the kinetics of drug release from nanofibers [32,33,34,35]. The Ritger–Peppas model is the most widely used model at present, which is used when the release data of the drugs is inconsistent with the Higuchi model. The release model fitting of nanofibers with different structures is shown in Table 2. PPR1, PPR2, and PPR3 fitted the Ritger–Peppas model the best, and PR1 and PR2 fitted the Higuchi model the best. The determination coefficients R^2^ of the five fitted equations were all greater than 0.95. The diffusion index n of the coaxial nanofibers in the Ritger–Peppas model were all less than 0.45, indicating that the drug release followed the Fick diffusion mechanism. The fitted curves of the nanofibers are shown in Supplementary Materials Tables S1 and S2. In summary, the release mechanism of ROX from the above nanofibers was mainly diffusion.

## 4. Conclusions

In summary, we fabricated coaxial porous PCL/PLA nanofibers using coaxial electrospinning and nonsolvent-induced phase separation successfully. The SEM images revealed that pores were distributed on the surface of nanofibers. FTIR showed that the antimicrobial agent ROX was loaded onto the PCL layer. Additionally, DSC and TGA indicated that the drugs that existed in the nanofibers reduced the thermal stability of the nanofibers. Compared with the coaxial nonporous nanofibers and nanofibers prepared by uniaxial electrospinning with or without pores, the prepared coaxial porous nanofibers slowed down the drug burst release phenomenon, promoted sustained release, and increased the solubility of hydrophobic drugs. Besides this, coaxial porous nanofibers encapsulated with ROX showed good antibacterial activity against *S. aureus*. Therefore, coaxial porous nanofibers might have potential applications in the sustained release of drugs.

## Figures and Tables

**Table 1:** Experimental parameters for the fabrication of the different nanofibers.

No.	Sample	Process	Solvent Type		Flow Rate (mL/h)		ROX Content ^3^ (%)
			Sheath ^1^	Core ^2^	Sheath	Core	
PPR1	PCL/PLA	Coaxial	CF/DMSO	CF/DMSO	0.2	0.1	15
PPR2	PCL/PLA	Coaxial	CF	CF/DMSO	0.2	0.1	15
PPR3	PCL/PLA	Coaxial	CF	TFE	0.2	0.1	15
PR1	PCL	Single	--	CF/DMSO	--	0.3	15
PR2	PCL	Single	--	TFE	--	0.3	15

^1^ The PLA concentration is 8% (*w*/*v*) and the CF/DMSO solvent ratio is 9:1 (*v*/*v*). ^2^ The PCL concentration is 10% (*w*/*v*) and the CF/DMSO solvent ratio is 9:1 (*v*/*v*). ^3^ The content of ROX 15% (*w*/*w*) is in respect to PCL.

**Table 2:** Drug release models of the different nanofibers.

No.	Model	Fitted Equation	R^2^	*n*
PPR1	zero-order model	M_t_/M_∞_ = 0.00502 + 0.00002 t	0.73113	0.1484
	the first-order model	ln (1 − Mt/M∞) = -0.00860 t − 0.59935	0.87218	
	Higuchi model	M_t_/M_∞_ = 0.00416 + 0.00032 t^0.5^	0.89865	
	Ritger-Peppas model	M_t_/M_∞_ = 0.00393 t^0.1484^	0.9876	
PPR2	zero-order model	M_t_/M_∞_ = 0.00477 + 0.00002 t	0.79783	0.14748
	the first-order model	ln (1 − M_t_/M_∞_) = −0.00671 t − 0.57268	0.92144	
	Higuchi model	M_t_/M_∞_ = 0.00396 + 0.00030 t^0.5^	0.95075	
	Ritger-Peppas model	M_t_/M_∞_ = 0.00375 t^0.14748^	0.99752	
PPR3	zero-order model	M_t_/M_∞_ = 0.00611 + 0.00001 t	0.92653	0.06311
	the first-order model	ln (1 − M_t_/M_∞_) = −0.00342 t − 0.91761	0.9654	
	Higuchi model	M_t_/M_∞_ = 0.00572 + 0.00016 t^0.5^	0.98022	
	Ritger-Peppas model	M_t_/M_∞_ = 0.00562 t^0.06311^	0.88921	
PR1	zero-order model	M_t_/M_∞_ = 0.00821+ 0.00001 t	0.87736	0.03243
	the first-order model	ln (1 − M_t_/M_∞_) = -0.00616 t − 1.65887	0.93903	
	Higuchi model	M_t_/M_∞_ = 0.00794 + 0.00010 t^0.5^	0.96394	
	Ritger-Peppas model	M_t_/M_∞_ = 0.00785 t^0.03243^	0.92104	
PR2	zero-order model	M_t_/M_∞_ = 0.00786 + 0.00001 t	0.8569	0.03431
	the first-order model	ln (1 − M_t_/M_∞_) = −0.00454 t − 1.5017	0.91754	
	Higuchi model	M_t_/M_∞_ = 0.00760 + 0.00010 t^0.5^	0.95155	
	Ritger-Peppas model	M_t_/M_∞_ = 0.00750 t^0.03431^	0.92593	

## Supplementary Materials

The following are available online at https://www.mdpi.com/article/10.3390/nano11051316/s1. Table S1: Fitted curves of coaxial nanofibers PPR1, PPR2, and PPR3. Table S2: Fitted curves of nanofibers PR1 and PR2 prepared by uniaxial electrospinning.
