# Supplementary material for: Antibacterial Porous Coaxial Drug-Carrying Nanofibers for Sustained Drug-Releasing Applications
Source: Nanomaterials (Basel). 2021 May 17;11(5):1316. doi: 10.3390/nano11051316 (PMC8157037; doi:10.3390/nano11051316)
Supplement: Supplementary file 1 [file nanomaterials-11-01316-s001.zip › nanomaterials-1150865-SI-done.pdf]

Antibacterial Porous Coaxial Drug-carrying Nanofibers for Sustained Drug-releasing Applications

Xin Chen [1,2], Honghai Li [1], Weipeng Lu [1,*], Yanchuan Guo [1,2,*]

[1] Key Laboratory of Photochemical Conversion and Optoelectronic Material, Technical Institute of Physics and Chemistry, Chinese Academy of Sciences, Beijing 100190, China; chenxin@mail.ipc.ac.cn (X.C.); lhh@mail.ipc.ac.cn (H.L.)
[2] Technical Institute of Physics and Chemistry, University of Chinese Academy of Sciences, Beijing 100049, China
* Correspondence: luweipeng@mail.ipc.ac.cn (W.L.); yanchuanguo@mail.ipc.ac.cn (Y.G.); Tel.: +86-010-8254-3445

Supporting Information

Table S1. Fitted curves of coaxial nanofibers PPR1, PPR2, PPR3.

	PPR1	PPR2	PPR3
zero-order model	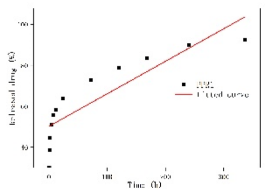	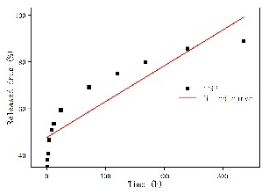	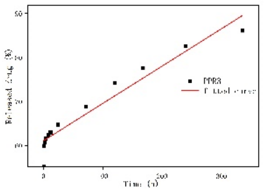
the first-order model	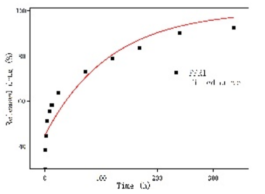	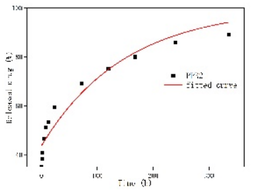	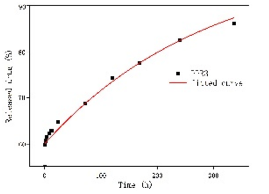
Higuchi model	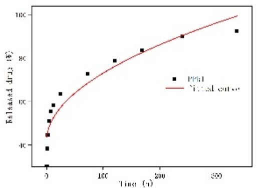	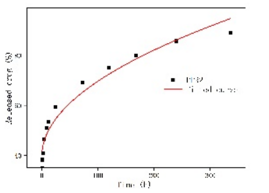	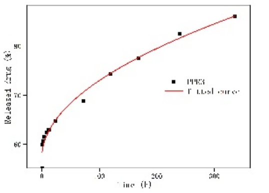
Ritger-Peppas model	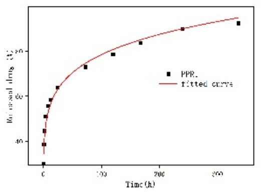	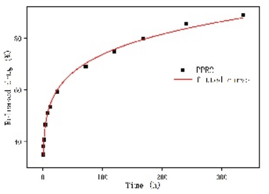	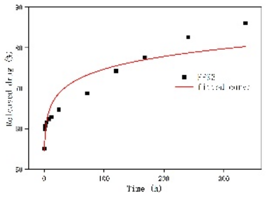

Table S2. Fitted curves of nanofibers PR1, PR2 prepared by uniaxial electrospinning.

	PR1	PR2
zero-order model	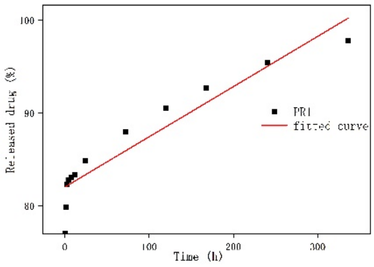	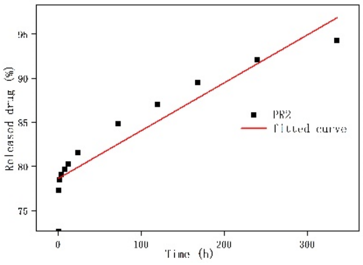
the first-order model	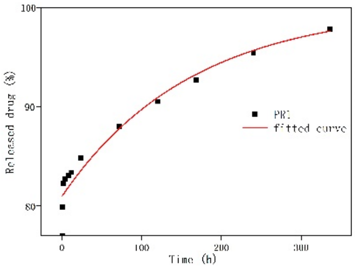	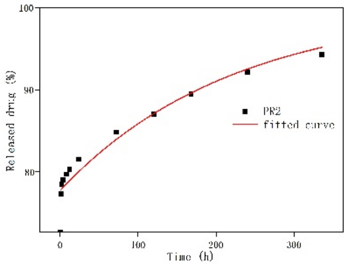
Higuchi model	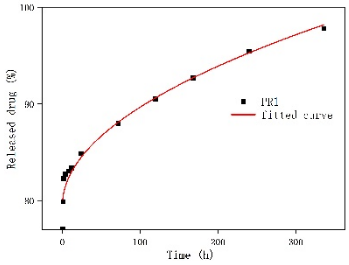	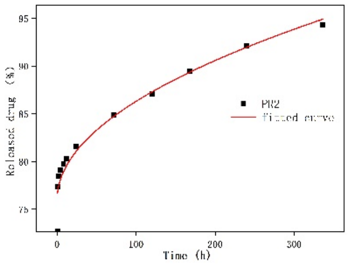
Ritger-Peppas model	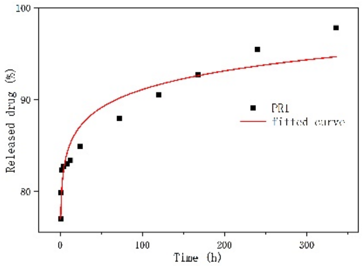	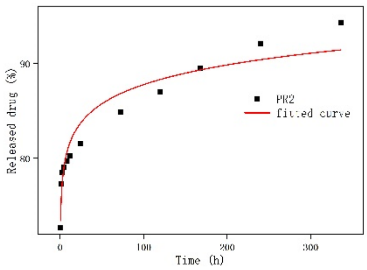
